# Genomic Risk Prediction of Incident Atrial Fibrillation in Older Individuals Without Prior Cardiovascular Disease

**DOI:** 10.1016/j.jacadv.2025.102245

**Published:** 2025-10-21

**Authors:** Peter Daniel Fransquet, Chenglong Yu, Cammie Tran, Sultana Monira Hussain, Johannes T. Neumann, Jocasta Ball, Tian Lin, Lawrence Beilin, Mark R. Nelson, Kevan R. Polkinghorne, Zhen Zhou, Diane Fatkin, Amy Brodtmann, Andrew Tonkin, John J. McNeil, Paul Lacaze

**Affiliations:** a School of Public Health and Preventive Medicine, Monash University, Melbourne, Victoria, Australia; b Victorian Heart Institute, Monash University, Clayton, Melbourne, Victoria, Australia; c University Medical Centre Hamburg, Department of Cardiology, Hamburg, Germany; d Department of Cardiology, Alfred Hospital, Melbourne, Victoria, Australia; e Monash Alfred Baker Centre for Cardiovascular Research, Monash University, Melbourne, Victoria, Australia; f Community Prevention and Cardiac Research, Baker Heart and Diabetes Institute, Melbourne, Victoria, Australia; g Institute of Molecular Bioscience, University of Queensland, St Lucia, Queensland, Australia; h Medical School, Royal Perth Hospital Unit, University of Western Australia, Perth, Australia; i Menzies Institute for Medical Research, University of Tasmania, Hobart, Australia; j Department of Nephrology, Monash Health, Clayton, Melbourne, Victoria, Australia; k Department of Medicine, Monash University, Clayton, Melbourne, Victoria, Australia; l Victor Chang Cardiac Research Institute, Darlinghurst, New South Wales, Australia; m School of Clinical Medicine, Faculty of Medicine and Health, UNSW Sydney, Kensington, New South Wales, Australia; n Cardiology Department, St Vincent’s Hospital, Darlinghurst, New South Wales, Australia; o Department of Neuroscience, School of Translational Medicine, Monash University, Melbourne, Victoria, Australia

**Keywords:** atrial fibrillation (AF), clinical risk prediction, genetic epidemiology, healthy aging, polygenic risk score (PRS)

## Abstract

**Background:**

Polygenic risk scores (PRSs) may enhance atrial fibrillation (AF) risk prediction when added to conventional risk factors. Most AF-PRS studies, however, focus on individuals with existing cardiovascular disease, rather than initially healthy older adults followed prospectively.

**Objectives:**

The objective of the study was to evaluate the predictive performance of a recent (2025) AF-PRS for incident AF in a cohort of healthy older individuals without prior cardiovascular events.

**Methods:**

AF-PRS was calculated in 12,906 individuals aged ≥65 years without prior cardiovascular disease or AF at enrollment into the ASPREE (Aspirin in Reducing Events in the Elderly) trial. Cox proportional hazards models assessed HRs) per SD of AF-PRS, alone and with clinical risk factors (age, sex, body mass index, hypertension, diabetes, dyslipidaemia, thyroid-stimulating hormone, smoking, and alcohol). We compared AF-PRS to clinical scores (Cohorts for Heart and Aging Research in Genomic Epidemiology [CHARGE]-AF and hypertension, age, raised body mass index, male sex, sleep apnea, smoking, and Alcohol score[HARMS2-AF]). Model performance was evaluated using Harrell’s C-index and likelihood ratio tests. Sex-stratified analysis was also conducted.

**Results:**

Over a median 4.5-years of follow-up, 654 incident AF cases occurred. AF-PRS was associated with incident AF (adjusted HR: 1.74 per SD; 95% CI: 1.58-1.84) (compared to CHARGE-AF [HR: 1.50] and HARMS2-AF [HR: 1.32] [all *P* < 0.0001]). Individuals in the highest AF-PRS quintile had 5.44-fold higher risk than those in the lowest (*P* < 0.0001). The AF-PRS showed stronger association in women than men (HR: 7.09 vs 4.51; interaction *P* = 0.007). AF-PRS improved prediction beyond clinical factors, increasing the C-index by 8.2% (0.63 → 0.71), 9.5% over CHARGE-AF (0.61 → 0.70), and 10.5% over HARMS2-AF (0.57 → 0.65).

**Conclusions:**

The use of an AF-PRS improves risk prediction of incident AF above clinical risk factors in older individuals without cardiovascular disease.

Atrial fibrillation (AF) is a common cardiac arrhythmia, affecting ∼60 million people worldwide.^1^ AF is associated with an increased risk of heart failure and cardioembolic stroke, cognitive impairment, and death.^2^, ^3^, ^4^ Common risk factors for AF include age, prior cardiovascular disease (CVD), hypertension, obesity, alcohol use, and family history.^2^ Widely used clinical risk scores such as the “Cohorts for Heart and Aging Research in Genomic Epidemiology Atrial Fibrillation” (CHARGE-AF) can be useful in predicting the AF risk. However, these equations show variable performance in different cohorts.^5^ A more accurate risk prediction of AF may help improve early detection, timely diagnosis, intervention and risk-management, especially in older individuals where AF prevalence is high.

Polygenic risk scores (PRSs) are an emerging tool for AF risk prediction, based on the aggregated effect of many different common genetic variants.^6^ The heritability (genetic component) of AF has been estimated at 22%.^7^ This relatively high genetic contribution suggests that genetic markers could play an important role in AF risk prediction. However, more validation studies of AF-PRS performance are required, to assess potential clinical utility.^8^ Most prior AF-PRS studies have been conducted in cohorts of individuals with existing CVD (eg, stroke and AF), rather than in initially-healthy individuals (without CVD) followed prospectively. This raises the question of how well the AF-PRS might perform in predicting incident AF (eg, for the setting of primary prevention), vs predicting risk in those already identified with disease.

Several prior AF-PRS studies have demonstrated that integrating a PRS with clinical risk factors and biomarkers can enhance AF risk prediction in individuals with existing CVD.^9^, ^10^, ^11^ In the present study, we assess the predictive performance of a recent AF-PRS^12^ in a longitudinal cohort of 12,906 initially healthy older individuals without diagnosed AF or CVD. The goal of the study is to ascertain whether the AF-PRS could improve risk prediction for incident AF in the setting of primary prevention, when added to conventional clinical risk factors, in older individuals without existing CVD.

## Methods

### Study population

**Figure 1:**
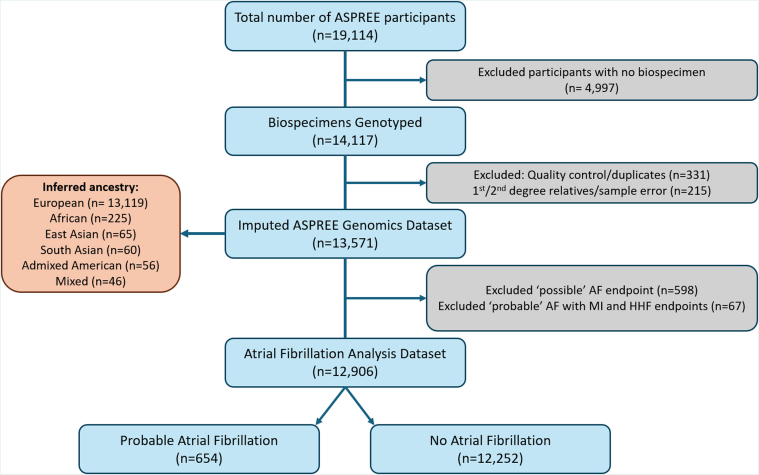
**Flowchart of Atrial Fibrillation Data Set Selection** AF = atrial fibrillation; ASPREE = Aspirin in Reducing Events in the Elderly; HHF = hospitalization for heart failure; MI = myocardial infarction.

This study included genotyped participants from the ASPREE (Aspirin in Reducing Events in the Elderly) trial, a randomized, double-blind, placebo-controlled trial for the effects of low-dose aspirin on disability-free survival in 19,114 older community-dwelling individuals from Australia and the United States.^13^ At inclusion, ASPREE participants were 70 years or older (US minorities ≥65 years old), relatively healthy, and without a history of diagnosed CVD events, AF, dementia, life-limiting physical disability, or illness that could limit survival to <5 years. All ASPREE participants had no history of diagnosed myocardial infarction (MI), heart failure, stroke, transient ischemic attack, AF, or systolic blood pressure > 180 mm Hg at enrollment. We conducted a post hoc genetic analysis of ASPREE participants who had both genomic data available and either probable AF, or no AF determined (n = 12,906) (Figure 1). All participants provided written informed consent. The study was approved by the Human Research Ethics Committees at Monash University and Alfred Hospital in Australia and site-specific institutional review boards in the United States, and registered on ClinicalTrials.gov (NCT01038583).

### AF endpoint

As published previously,^14^^,^^15^ AF in ASPREE was identified using a probabilistic algorithm incorporating AF medication and clinical evidence. Designated categories were *probable* AF, *possible* AF, and *no* AF. Participants were considered to have “probable AF” when a new AF diagnosis was identified within clinical notes or via self-report of a new AF diagnosis supported by a new prescription of any anticoagulant (ATC = B01A) or in a new prescription of digoxin (ATC = C01A) or an AF antiarrhythmic (ATC = C01B; eg, amiodarone) in the trial documentation (n = 721). Probable AF cases were substantiated by clinical evidence via ECG reports, admissions documenting AF, or correspondence from specialists stating AF had been detected. Participants were assigned “possible AF” if they had indications in the trial documentation that they possible had AF, but the evidence was not substantiated, thus not strong enough for classification as probable AF (n = 598). The “no AF” group were participants without any AF triggers (n = 12,252).

Cases of MI or hospitalization for heart failure that occurred before or simultaneously with a recorded probable AF case (n = 67) were excluded from the analysis, leaving a total of 654 probable incident AF cases by follow-up. For higher specificity of AF diagnosis, participants with possible AF were excluded. The primary analysis was carried out using data from the probable AF group only, comparing to the no AF group.

### Genetic analysis and polygenic risk score

DNA collected at baseline was used to genotype ∼850,000 genetic variants using the Axiom 2.0 Precision Medicine Diversity Array (Thermo Fisher Scientific). A custom pipeline was used for variant calling which aligned data to the human reference genome GRCh38 (hg38). Quality control (QC) removed low quality samples and single nucleotide polymorphisms.^16^ Genetic ancestry and relationships were inferred^17^ and imputation was performed using the TOPMed Imputation Server with the TOPMed ‘r3’ reference panel, containing 133,597 genetically diverse reference samples with data from over 445 million genetic variants.^18^, ^19^, ^20^ Variants were removed in postimputation QC if imputation quality score R2 was <0.3.

The multiancestry AF-PRS from Roselli et al. was generated from a large meta-analysis of 68 summary-level results from more than 40 primary cohorts which included 181,446 AF cases and 1,468,899 controls.^12^ Variant weights were retrieved from the Cardiovascular Disease Knowledge Portal using the weblink https://cvd.hugeamp.org/downloads.html#polygenic, as per the data availability statement. This AF-PRS uses 1,113,668 variants, of which 1,093,502 were available in the ASPREE genetic dataset after QC. Plink 1.9 was used to generate the AF-PRS.^21^

### Clinical risk scores

CHARGE-AF is a widely used, 5-year clinical risk score built using AF risk variables from 3 large U.S. cohorts.^11^ CHARGE-AF is scored on a continuous scale, which uses the following weighted variables in its calculation; age, Caucasian ancestry, height, weight, systolic and diastolic blood pressure, hypertension treatment, current smoking, diabetes, and history of heart failure and MI. The coefficients of these variables used for calculation are in Supplemental File 1.

For calculation of CHARGE-AF in ASPREE participants, we grouped those with inferred non-Finnish European genetic ancestry as “White”, and all else as “not White”. There were no participants with Finnish ancestry in this study. Systolic and diastolic blood pressure were derived by using the average of 3 baseline blood pressure measurements from ASPREE. For hypertension treatment, we classified any participant as “yes” if on 1 or more of the following were prescribed in the baseline year: any agents acting on the renin-angiotensin system, beta blocking agents, calcium channel blockers, or diuretics. Due to ASPREE’s trial exclusion criteria, no participant had a history of hospitalization for heart failure or MI at baseline.

The hypertension, age, raised BMI, male sex, sleep apnea, smoking and alcohol (HARMS2-AF) score was derived using AF risk factors from 314,280 participants of the U.K. Biobank, of which 17,914 were incident cases of AF over a median follow-up of 12.9 years.^22^ HARMS2-AF is scored between 0 and 14, calculated using the following variables: hypertension, age, body mass index (BMI), sex, sleep apnea, smoking (current/previous), and standard alcoholic drinks per week. Coefficients used for calculation are detailed in Supplemental File 1.

In ASPREE, sleep apnea data were available for only a small subset (n = 1,399),^23^ thus all ASPREE participants were allocated a sleep apnea score of 0 in this study. The specific number of alcoholic drinks per week were not recorded in ASPREE; thus in this study, drinks per week were derived from the average of reported days per week (as it was reported as 1-2 days, 3-4 days, 5-6 days or everyday) multiplied by the number of average drinks per occasion.

### Statistical analysis

Cox proportional hazard regression models estimated associations between the AF-PRS and AF events in ASPREE, both as a standardized continuous AF-PRS variable (HR per SD) and categorically in quintiles, where AF-PRS quintile 1 was the reference group. Comparisons between quintiles were carried out using the chi-squared test (categorical data) or analysis of variance/*t*-test (continuous data). The basic model (model 1) included the standardized AF-PRS, age, sex, and the first 10 genetic principal components. Subsequent models included further adjustments based on previous AF studies within ASPREE. Model 2 adjusts for variables in the model 1, plus hypertension, diabetes, BMI, and alcohol use (current vs previous/never) based on Ball et al.^14^ Model 3 adjusts for model 2 variables, plus smoking (current vs previous/never), dyslipidaemia, thyroid-stimulating hormone (TSH), and aspirin allocation, based on Tran et al.^15^

Details of baseline data collection are provided in Supplemental File 1. As ASPREE was a randomized control trial for aspirin use, we assessed the interaction between AF and aspirin treatment allocation. We also examined sex-interaction and sex stratification for all 3 models, due to known differences in AF incidence by sex.^24^

CHARGE-AF and HARMS2-AF were also analyzed using Cox proportional hazard regression models, both with the clinical risk score alone, and then with the AF-PRS and first 10 principal components. Sex stratification was undertaken for CHARGE-AF but not HARMS2-AF as HARMS2-AF uses sex in its scoring. Model comparisons were carried out using Harrell's C-index to compare each predictor alone, as well as when including the AF-PRS, and statistical differences between models were assessed using likelihood ratio tests. All statistical analyses were performed using R (version 4.3.0, R Core Team 2024).

## Results

### Baseline characteristics

Baseline characteristics for the 12,906 participants in this study are shown in Table 1. Characteristics are displayed for all participants, as well as stratified by quintiles of polygenic risk (quintile 1 being the lowest risk for AF and quintile 5 being the highest risk). The mean age of participants was 74.9 years (SD 4.2) (median:73.8, range:65.1-96), and 7,121 (55.2%) were women. When stratifying AF-PRS risk over quintiles, age and sex were significantly different between groups. Values for BMI and TSH were missing for some participants (n = 56 and n = 1,664, respectively).

**Table 1:** Baseline Participant Characteristics Stratified by Polygenic Risk Quintiles

	Overall (N = 12,906)	Quintile 1 (n = 2,582)	Quintile 2 (n = 2,581)	Quintile 3 (n = 2,581)	Quintile 4 (n = 2,581)	Quintile 5 (n = 2,581)	*P* Value^a^
Age, y, mean (SD)	74.9 (4.2)	75.3 (4.3)	75.0 (4.2)	74.9 (4.4)	75.0 (4.3)	74.7 (4.2)	<0.0001
Sex ♀, n (%)	7,121 (55.2)	1,400 (54.2)	1,387 (53.7)	1,410 (54.6)	1,422 (55.1)	1,502 (58.2)	0.011
BMI^b^, mean (SD)	28.0 (4.6)	27.8 (4.5)	28.0 (4.6)	27.9 (4.4)	28.2 (4.6)	28.1 (4.6)	0.051
Hypertension, n (%)	9,483 (73.5)	1896 (73.4)	1887 (73.1)	1882 (72.9)	1915 (74.2)	1904 (73.8)	0.850
Diabetes, n (%)	1,244 (9.6)	242 (9.4)	250 (9.7)	264 (10.2)	245 (9.5)	243 (9.4)	0.833
Dyslipidaemia, n (%)	8,543 (66.2)	1712 (66.3)	1715 (66.5)	1714 (66.4)	1707 (66.1)	1,696 (65.7)	0.977
TSH (mU/L)^c^, mean (SD)	1.6 (2.0)	1.6 (2.1)	1.6 (1.8)	1.6 (1.5)	1.6 (2.8)	1.6 (1.9)	0.720
Smoking status							
Current, n (%)	404 (3.1)	73 (2.8)	73 (2.8)	87 (3.4)	76 (2.9)	95 (3.7)	0.432
Former, n (%)	5,299 (41.1)	1,034 (40.0)	1,061 (41.1)	1,069 (41.4)	1,053 (40.8)	1,083 (41.9)	
Never, n (%)	7,203 (55.8)	1,475 (57.1)	1,447 (56.1)	1,425 (55.2)	1,452 (55.2)	1,404 (54.4)	
Alcohol use							
Current, n (%)	10,180 (78.9)	2038 (78.9)	2031 (78.7)	2077 (80.5)	2049 (79.4)	1985 (76.9)	0.432
Former, n (%)	659 (5.1)	141 (5.5)	126 (4.9)	117 (4.5)	140 (5.4)	135 (5.2)	
Never, n (%)	2067 (16.0)	403 (15.6)	424 (16.4)	387 (15.0)	392 (15.2)	461 (17.9)	
Aspirin, n (%)	6,471 (50.1)	1,316 (51.0)	1,299 (50.3)	1,297 (50.2)	1,280 (50.4)	1,279 (49.6)	0.841

BMI = body mass index; TSH = thyroid-stimulating hormone.

a To compare variables across quintile groups, chi-square tests were used for categorical variables, and ANOVA for continuous variables.

b 56 missing values.

c 1,664 missing values.

### Polygenic risk for AF

The median follow-up for the study period was 4.5 years, and a total of 654 (5.1%) participants reached a probable AF endpoint. Results of Cox proportional hazard models of the AF-PRS, as a continuous variable and categorically by quintile, can be seen in Table 2. Model 1 used all participants (n = 12,906); however, model 2 (n = 12,850) and model 3 (n = 11,198) featured slightly less due to missingness of BMI and TSH data.

**Table 2:** Cox Proportional Hazard Analysis for Atrial Fibrillation PRS (Continuous and Categorical)

AF-PRS	Model 1 (n = 12,906)		Model 2 (n = 12,850)^a^		Model 3 (n = 11,198)^a^	
	HR (95% CI)	*P* Value	HR (95% CI)	*P* Value	HR (95% CI)	*P* Value
Continuous	1.71 (1.59-1.84)	<0.0001	1.71 (1.58-1.84)	<0.0001	1.74 (1.61-1.89)	<0.0001
Categorical						
Q1	Reference		Reference		Reference	
Q2	2.03 (1.43-2.87)	<0.0001	2.02 (1.43-2.86)	<0.0001	1.91 (1.32-2.80)	0.0006
Q3	2.73 (1.95-3.81)	<0.0001	2.74 (1.96-3.82)	<0.0001	2.66 (1.87-3.79)	<0.0001
Q4	3.67 (2.66-5.07)	<0.0001	3.63 (2.63-5.01)	<0.0001	3.59 (2.55-5.06)	<0.0001
Q5	5.39 (3.94-7.37)	<0.0001	5.33 (3.89-7.28)	<0.0001	5.44 (3.91-7.56)	<0.0001

Model 1 = age, sex, and top 10 genetic principal components.

Model 2 = model 1 + hypertension, diabetes, body mass index, and alcohol intake (current/former vs never).

Model 3 = model 2 + smoking (current/former vs never), dyslipidemia, thyroid stimulating hormone and aspirin allocation.

AF-PRS = atrial fibrillation polygenic risk score; Q = quintile.

a Lower n due to missingness in variable data.

The AF-PRS was significantly associated with AF risk in all models, across both continuous and categorical analyses (fully adjusted continuous model 3: adjusted HR: 1.74; 95% CI: 1.61-1.89). When compared with the lowest risk quintile, the HRs increased in all cases across the models. Those at the highest genetic risk (quintile 5) had a 5.44-fold increased AF risk when compared to the lowest quintile (model 3, HR: 5.44; 95% CI: 3.91-7.56, *P* < 0.0001).

**Figure 2:**
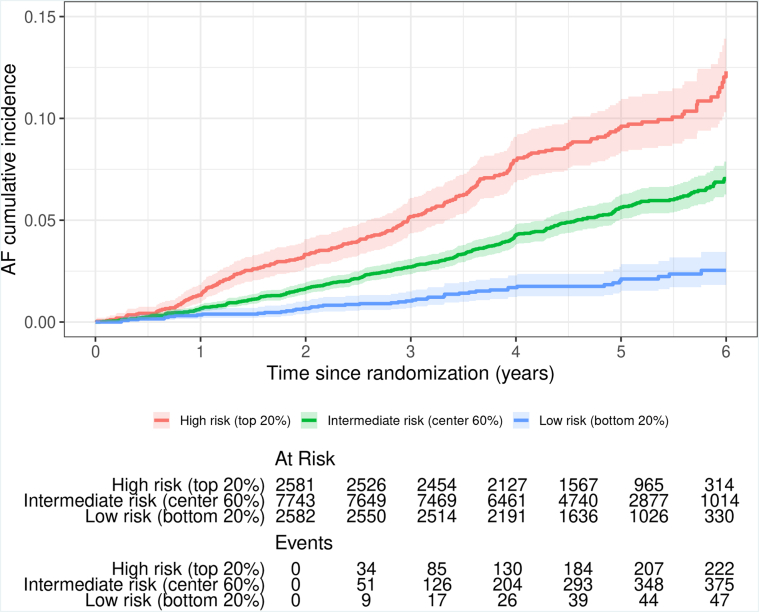
**6-Year Cumulative Incidence of Atrial Fibrillation, Stratified by Low (Quintile 1), Intermediate (Quintiles 2-4), and High (Quintile 5) Genetic Risk, Adjusted for Censoring Over Time** Abbreviations as in Figure 1.

Cumulative incidence of AF stratified by the lowest (quintile 1, intermediate (quintiles 2-4) and highest (quintile 5) genetic risk over a 6-year period can be seen in Figure 2. There was no interaction between AF-PRS and aspirin treatment group detected (Supplemental Table 1).

### Sex stratification of AF-PRS

We detected an interaction between the AF-PRS and sex (model 3, *P* = 0.007, other data not shown). Results for sex-stratification analysis over the 3 AF-PRS models can be seen in Supplemental Table 2. In the fully adjusted model, women (n = 327 probable AF, n = 7,121 no AF, HR: 1.90; 95% CI: 1.70-2.11; *P* < 0.0001) had a stronger association between the AF-PRS and incident AF risk compared to men (n = 327 probable AF, n = 5,785 no AF, HR: 1.56; 95% CI: 1.50-4.05; *P* < 0.0001).

When dividing into quintiles, women in the highest genetic risk quintile had 7.09 times more risk than the lowest risk quintile (95% CI: 4.36-11.55; *P* < 0.0001), whereas men had 4.51 times the risk when comparing the highest to lowest quintiles (95% CI: 2.82-7.20; *P* < 0.0001).

### AF-PRS vs CHARGE-AF and HARMS2-AF clinical risk scores

Characteristics used in the generation of CHARGE-AF and HARMS2-AF can be seen in Supplemental Tables 3 and 4, respectively. Due to missing data, CHARGE-AF was limited to 11,139 participants, and HARMS2-AF to 10,794. All characteristics used in the generation of CHARGE-AF, apart from diastolic blood pressure, smoking, and diabetes status, were significantly different (*P* < 0.05) between probable AF (n = 574) and no AF (n = 10,565). For HARMS2-AF, only estimated weekly standard drinks was not significant between probable AF (n = 550) and no AF (n = 10,244), although it showed a trend (*P* = 0.054). When used in Cox models, CHARGE-AF (HR: 1.50; 95% CI: 1.38-1.63;*P* < 0.0001) showed higher HR than HARMS2-AF (HR: 1.32; 95% CI: 1.21-1.44; *P* < 0.0001) (Supplemental Table 5). The basic AF-PRS model 1 showed higher HR than both scores (HR: 1.71; 95% CI: 1.59-1.84; *P* < 0.0001).

C-index estimates comparing all models and clinical risk scores with and without the AF-PRS can be seen in Table 3. Without the AF-PRS, models showed low to moderate discrimination between probable AF and no AF, with the C-index for the fully adjusted model (model 3) without AF-PRS being 0.63. For the CHARGE-AF score alone, the C-index was 0.61. For the HARMS2-AF score alone, the C-index was 0.57.

**Table 3:** C-Index Estimates for AF Risk Models With and Without AF-PRS

	C-Index (SE)	*P* Value^a^
Risk Factor Cox Model		
Cox model 3 (risk factors alone)	0.626 (0.011)	
Cox model 3 with AF-PRS	0.708 (0.010)	
C-index Δ	+8.2%	<0.0001
CHARGE-AF		
CHARGE-AF alone	0.607 (0.012)	
CHARGE-AF with AF-PRS, PCs & sex	0.702 (0.011)	
C-index Δ	+9.5%	<0.0001
CHARGE-AF in women		
CHARGE-AF alone	0.606 (0.016)	
CHARGE-AF with AF-PRS & PCs	0.716 (0.014)	
C-Index Δ	+11%	<0.0001
CHARGE-AF in men		
CHARGE-AF alone	0.620 (0.018)	
CHARGE-AF with AF-PRS & PCs	0.686 (0.016)	
C-index Δ	+6.6%	<0.0001
HARMS2-AF		
HARMS_2_-AF alone	0.572 (0.012)	
HARMS_2_-AF with AF-PRS & PCs	0.677 (0.011)	
C-index Δ	+10.5%	<0.0001

CHARGE-AF = Cohorts for Heart and Aging Research in Genomic Epidemiology Atrial Fibrillation; HARMS2-AF = hypertension, age, raised BMI, male sex, sleep apnea, smoking, and alcohol score; PCs = principal components; other abbreviations as in Table 2.

a From likelihood ratio test.

Adding the AF-PRS to the fully adjusted risk model increased the C-index by 8.2% (from 0.63 to 0.71). The AF-PRS also increased the C-index of existing clinical risk scores, including for CHARGE-AF increasing the C-index by 9.5% (from 0.61 to 0.70), and for HARMS2-AF by 10.5% (from 0.57 to 0.68).

When stratifying by sex, there was a difference in HR between women and men in CHARGE-AF (HR: 1.43 and HR: 1.58, respectively) (Supplemental Table 6). When including AF-PRS to CHARGE-AF in women only, C-index increased by 11% (0.61-0.72, *P* < 0.0001), and in men only, increased by 6.6% (0.62-0.69, *P* < 0.0001) (Table 3).

## Discussion

In this study, we assessed the performance of an AF-PRS derived from the largest-ever meta-analysis of genome-wide association studies for AF, in a population of initially healthy older individuals (without prior diagnosed AF or CVD) followed prospectively. A summary of this study can be seen in the Central Illustration. We found that the AF-PRS improved prediction of incident AF, above clinical risk factors and the commonly used AF risk prediction scores. We found that individuals in the highest AF-PRS quintile were 5.44 times more likely to develop incident AF than those in the low-risk quintile. The AF-PRS performed well in both men and women, with a stronger effect observed in women. In fully adjusted models, the AF-PRS showed substantial improvements in the C-index, with 9.5% and 10.5% improvements over CHARGE-AF and HARMS2-AF respectively. Our study provides new evidence toward a growing body suggesting that the addition of genomic and other biomarker measures could substantially improve the risk prediction for AF.^5^

**Central Illustration:**
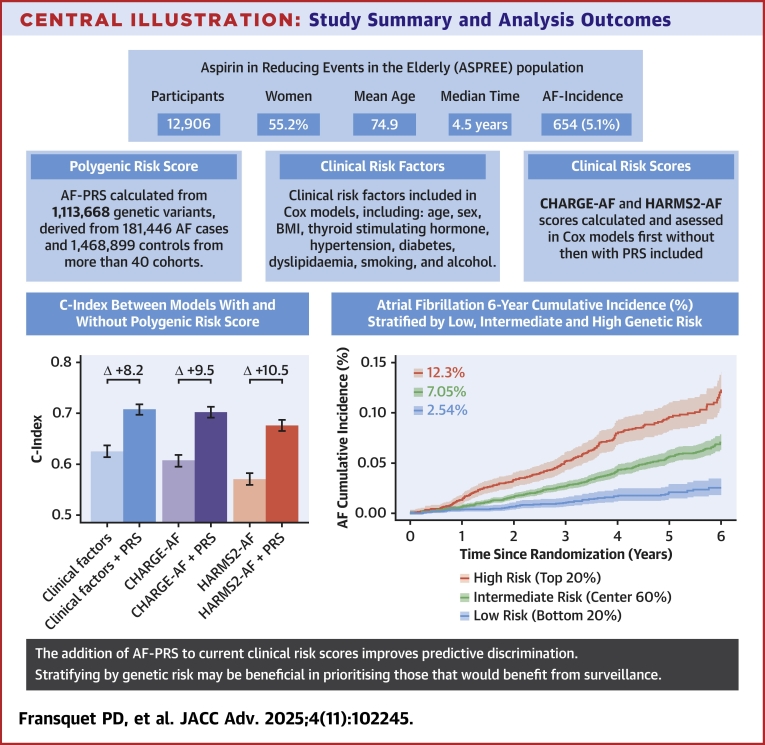
**Study Summary and Analysis Outcomes** The addition of AF-PRS to current clinical risk scores improves predictive discrimination in initially healthy older adults. Stratifying by genetic risk may be beneficial in prioritizing those what would benefit from surveillance. AF = atrial fibrillation; ASPREE = Aspirin in Reducing Events in the Elderly; CHARGE-AF = Cohorts for Heart and Aging Research in Genomic Epidemiology Atrial Fibrillation; HARMS2-AF = hypertension, age, raised BMI, male sex, sleep apnea, smoking, and alcohol score; PRS = polygenic risk score.

A previous study by Marston et al. used an older (2018) AF-PRS, alongside CHARGE-AF^9^ in 36,662 participants and observed a nearly 7-fold increase in risk when comparing the lowest vs highest AF-PRS quintiles (1.3% risk in low vs8.7% in high). Furthermore, when adding the biomarker N-terminal pro–B-type natriuretic peptide (NT-proBNP), model discrimination for AF was increased further (CHARGE-AF alone C-index: 0.65, and including NT-proBNP and AF-PRS C-index: 0.70). However, all participants included in this study had prior diagnosed MI (82.5%) or CVD. Another study of 49,881 participants generated an AF-PRS in individuals of European descent from the U.K. Biobank,^25^ and showed that those with a high genetic risk (>95th percentile) compared to those with low (<95th percentile) had a 2.42-fold increased risk of AF (95% CI: 2.33-2.52; *P* < 0.0001). Although the cohort did not specifically include individuals with CVD, the AF-PRS was generated only for Caucasians and used the 95th percentile as a cutoff rather than quintiles. These studies provide evidence that incorporating an AF-PRS alongside clinical risk factors improves risk prediction in individuals with existing disease, with key differences to our study.

In our study of the ASPREE trial cohort, in fully adjusted models we found substantially higher HRs and greater improvements in the C-index compared to these prior studies. This may be due to the depletion of the most common AF risk factors in the ASPREE study population at baseline, or due to other reasons. A particularly striking result was that women in the highest AF-PRS quintile were 7.09 times more likely to develop incident AF than those in the lowest AF-PRS quintile. Furthermore, the addition of the AF-PRS to CHARGE-AF in women only showed an 11% increase in C-index, compared to men which had a smaller but also substantial increase (6.6%). Conversely, another recent study showed that adding a PRS to CHARGE-AF increased the area under the curve significantly in men (from 0.64 to 0.75) but not in women (from 0.71 to 0.73).^26^ Sex differences in AF are notable, with women having a lower overall prevalence of AF than men, but often presenting with more severe AF symptoms, higher risk of AF-related stroke, and worse quality of life compared to men.^24^ Thus, our AF-PRS analysis may be of particular interest for interpreting AF risk in healthy older women, regarding possible intervention and prevention strategies. Performance of the AF-PRS was also strong in men, with men in the highest AF-PRS quintile at 4.51 times higher risk of incident AF than men in the lowest quintile.

Strengths of our study include the prospective study design, involving healthy older people followed over median 4.5 years, without prior diagnosed AF or CVD at baseline. This study design enabled the assessment of incident AF, rather than prevalent disease. Furthermore, the categorization and assessment of AF as an outcome in our study was supported by rigorous methodology and clinical evidence review within the ASPREE trial, as published previously.^14^^,^^15^ Our analysis did not rely on self-report, International CLassification of Diseases-10 codes or other ascertainment methods for AF, but on a combination of multiple factors and clinical evidence. Our study provides a timely external validation of a newly derived (2025) AF-PRS, in an independent prospective cohort, that was not part of the initial discovery genome wide association study. Our study also provides a unique contribution for the assessment of AF risk specifically in the setting of primary prevention in older people.

### Study Limitations

Limitations of our study include the predominance of European ancestry in the study population (96.6%), limiting the generalizability of our findings to other genetic ancestries. We note the 2025 Roselli AF-PRS is a multiancestry AF-PRS, derived from more diverse cohorts than previous AF-PRSs. Further studies are warranted to assess the performance of the AF-PRS in more diverse populations. Given the generally healthy status of the ASPREE cohort at baseline, our findings may reflect an enrichment of genetic AF risk in the absence of significant clinical risk factors (aside from age). Furthermore, having reached older age with good overall health, it is also possible that individuals in the ASPREE cohort are enriched for protective genetic factors for common comorbid conditions, potentially leading to an overestimation of AF genetic risk effects compared to populations with greater clinical burden. Thus, the generalizability of our findings to older adults with more prevalent comorbidities remains uncertain. Future studies comparing AF-PRS performance across more diverse cohorts with varying baseline health status—and accounting for both risk-enhancing and protective genetic variation—are needed to assess the relative predictive utility of genetic vs clinical risk factors. Another limitation was missing variables for calculation of the clinical risk scores CHARGE-AF and HARMS2-AF, including sleep apnea. Our study did not include biomarker data, particularly NT-proBNP, which has great potential for improving AF risk prediction when added to clinical risk scores.^9^^,^^27^ Using other “omics” alongside AF-PRS may increase the accuracy of predictive risk models, particularly in the exploration of new biomarkers.^28^, ^29^, ^30^

In conclusion, our study demonstrates an improved risk prediction for incident AF when adding a PRS to conventional risk factors, in a cohort of initially healthy older individuals followed prospectively. Our study provides new evidence to suggest a future role for genomic risk prediction in the early identification and primary prevention of AF.

Perspectives **COMPETENCY IN PATIENT CARE AND PROCEDURAL SKILLS:** This study demonstrates that the use of a PRS significantly improves risk prediction for incident AF in older adults, beyond conventional clinical risk factors alone. The findings have relevance to Medical Knowledge and Patient Care, by supporting the early identification and risk management of individuals at high risk of AF, who might benefit from more targeted screening, closer monitoring, or tailored interventions. The study highlights the use of genomics as a potential new tool for enabling more proactive preventive strategies to reduce the burden of AF and related complications, such as stroke and heart failure.

**TRANSLATIONAL OUTLOOK:** Although these findings support the possible clinical utility of genomic risk prediction for AF, key barriers to translation of genomics in primary care exist, including the need for further validation across diverse ancestry groups, and cost-effectiveness evaluation. Future research from large, prospective studies will help guide the potential implementation of PRS-guided screening, and inform the current discussion around clinical utility. Ongoing efforts are required to address equity in genomic medicine, ensuring that benefits of such tools would be accessible across varied populations.

## Funding support and author disclosures

The ASPREE (ASPirin in Reducing Events in the Elderly) trial Biobank is supported by a Flagship cluster grant (including the Commonwealth Scientific and Industrial Research Organisation, Monash University, Menzies Research Institute, Australian National University, University of Melbourne); and a grant (5U01AG29824-02) from the 10.13039/100000054National Cancer Institute at the National Institutes of Health; and 10.13039/501100001779Monash University. The ASPREE project is supported by grants (U01AG029824 and U19AG062682) from the 10.13039/100000049National Institute on Aging and the 10.13039/100000054National Cancer Institute at the National Institutes of Health; and by grants (334047 and 1127060) from the 10.13039/501100000925National Health and Medical Research Council of Australia; and by Monash University and the 10.13039/100008018Victorian Cancer Agency. P.L. is supported by a National Heart Foundation Future Leader Fellowship (107171) and 10.13039/501100000925National Health and Medical Research Council of Australia Investigator Grant (2026325). Dr Yu is supported by a Vanguard Grant (108071-2024_VG) from the 10.13039/501100001030National Heart Foundation of Australia. All other authors have reported that they have no relationships relevant to the contents of this paper to disclose.
