# Supplementary material for: Genomic Risk Prediction of Incident Atrial Fibrillation in Older Individuals Without Prior Cardiovascular Disease
Source: JACC Adv. 2025 Oct 21;4(11):102245. doi: 10.1016/j.jacadv.2025.102245 (PMC12589920; doi:10.1016/j.jacadv.2025.102245)
Supplement: Supplementary material [file mmc1.docx]

**Supplementary Tables for “Genomic risk prediction of incident atrial fibrillation in older individuals without prior cardiovascular disease”**Table S1. AF-PRS and aspirin interaction

|  | **Aspirin x AF-PRS (Model 3)** | |
| --- | --- | --- |
| AF_PRS | **HR (95% CI)** | **p-value** |
| Continuous*Aspirin | 1.08 (0.92 - 1.26) | 0.36 |
| Categorical |  |  |
| Q1*Aspirin | Reference | |
| Q2*Aspirin | 0.97 (0.46 – 2.04) | 0.94 |
| Q3*Aspirin | 1.06 (0.52 – 2.15) | 0.87 |
| Q4*Aspirin | 0.75 (0.38 – 1.48) | 0.40 |
| Q5*Aspirin | 1.16 (0.60 – 2.25) | 0.65 |

AF-PRS, Atrial Fibrillation Polygenic Risk Score; CI, Confidence Interval; HR, Hazard Ratio, Q, Quintile.

Table S2. Sex Stratification Cox models analysis for atrial fibrillation PRS (continuous and categorical)

|  | **Model 1** | | | |
| --- | --- | --- | --- | --- |
|  | **Women  (n=327 probable AF, n=7121 no AF)** | | **Men**  **(n=327 probable AF, n=5785 no AF)** | |
|  | **HR (95% CI)** | **p-value** | **HR (95% CI)** | **p-value** |
| Continuous (per SD) | 1.90 (1.70 - 2.11) | <0.0001 | 1.56 (1.40 – 1.73) | <0.0001 |
| Categorical |  |  |  |  |
| Q1 | Reference | | | |
| Q2 | 1.71 (1.04 – 2.81) | 0.04 | 2.47 (1.50 – 4.05) | 0.0004 |
| Q3 | 2.47 (1.55 – 3.95) | 0.0002 | 3.03 (1.86 – 4.92) | <0.0001 |
| Q4 | 3.35 (2.13 – 5.28) | <0.0001 | 4.28 (2.68 – 6.84) | <0.0001 |
| Q5 | 5.97 (3.88 – 9.19) | <0.0001 | 5.00 (3.14 – 7.94) | <0.0001 |
|  | **Model 2** | | | |
| Continuous (per SD) | 1.89 (1.70 – 2.10) | <0.0001 | 1.56 (1.40 – 1.73) | <0.0001 |
| Categorical |  |  |  |  |
| Q1 | Reference | | | |
| Q2 | 1.69 (1.03 – 2.78) | 0.04 | 2.49 (1.52 – 4.09) | 0.0003 |
| Q3 | 2.48 (1.55 – 3.96) | 0.0002 | 3.07 (1.89 – 5.00) | <0.0001 |
| Q4 | 3.30 (2.10 – 5.19) | <0.0001 | 4.25 (2.66 – 6.81) | <0.0001 |
| Q5 | 5.95 (3.87 – 9.16) | <0.0001 | 4.97 (3.13 – 7.91) | <0.0001 |
|  | **Model 3** | | | |
| Continuous (per SD) | 1.99 (1.77 – 2.23) | <0.0001 | 1.54 (1.38 – 1.71) | <0.0001 |
| Categorical |  |  |  |  |
| Q1 | Reference | | | |
| Q2 | 1.74 (0.98 – 3.09) | 0.057 | 2.26 (1.36 – 3.74) | 0.002 |
| Q3 | 2.74 (1.61 – 4.66) | 0.0002 | 2.64 (1.61 – 4.35) | 0.0001 |
| Q4 | 3.84 (2.30 – 6.42) | <0.0001 | 3.73 (2.31 – 6.00) | <0.0001 |
| Q5 | 7.09 (4.36 – 11.55) | <0.0001 | 4.51 (2.82 – 7.20) | <0.0001 |

Model 1 = age, sex, and top 10 genetic principal components;
Model 2 = model 1 + hypertension, diabetes, body mass index, and alcohol intake (current/former vs never);
Model 3 = model 2 + smoking (current/former vs never), dyslipidaemia, thyroid stimulating hormone and aspirin allocation
AF, Atrial Fibrillation; CI, Confidence Interval; HR, Hazard Ratio, Q, Quintile.

Table S3. Baseline characteristics for CHARGE-AF clinical risk score

| **Baseline characteristics*** | **Incident AF**  **(n=574)** | **No AF**  **(n=10,565)** | **p**-**value**** |
| --- | --- | --- | --- |
| **Age, mean (SD)** | 76.4 (4.6) | 75 (4.2) | <0.0001 |
| **White, n (%)** | 564 (98.3) | 10196 (96.5) | 0.032 |
| **Height (cm), mean (SD)** | 167.3 (9.6) | 165.1 (9.2) | <0.0001 |
| **Weight (kg), mean (SD)** | 80.5 (16.1) | 77.0 (14.7) | <0.0001 |
| **Systolic BP (mmHg), mean (SD)** | 141 (17.4) | 139.2 (16.2) | 0.016 |
| **Diastolic BP (mmHg), mean (SD)** | 77.2 (10.6) | 77.1 (9.9) | 0.74 |
| **Hypertension Treatment, n (%)** | 390 (68.0) | 6152 (58.2) | <0.0001 |
| **Smoking (Current), n (%)** | 14 (2.4) | 334 (3.2) | 0.40 |
| **Diabetes, n (%)** | 66 (11.5) | 1139 (10.8) | 0.64 |
|  |  |  |  |
| **CHARGE-AF, mean (SD)** | 110.31 (5.2) | 108.23 (5.0) | <0.0001 |

BP, blood pressure; SD, standard deviation.
*There were no cases of heart failure or myocardial infarction at baseline, limited to n=11,139 due to missing data.
** To compare variables across case/control groups, chi-square tests were used for categorical variables, and t-test for continuous variables.

Table S4. Baseline characteristics for HARMS2-AF clinical risk score

| **Baseline characteristics*** | **Incident AF**  **(n=550)** | **No AF**  **(n=10,244)** | **p-value**** |
| --- | --- | --- | --- |
| **Age, mean (SD)** | 76.2 (4.6) | 74.8 (4.2) | <0.0001 |
| **BMI, mean (SD)** | 28.4 (4.7) | 27.9 (4.4) | 0.0046 |
| **Sex (Male), n (%)** | 293 (53.3) | 4953 (48.4) | 0.027 |
| **Hypertension, n (%)** | 447 (81.3) | 7434 (72.6) | <0.0001 |
| **Smoking (Current/Previous), n (%)** | 306 (55.6) | 4988 (48.7) | 0.0017 |
| **Alcohol, n (%)** |  |  |  |
| <7 std. p/w | 299 (54.4) | 6038 (58.9) | 0.054 |
| 7-14 std. p/w | 171 (31.1) | 3001 (29.3) |  |
| ≥15 std. p/w | 80 (14.5) | 1205 (11.8) |  |
|  |  |  |  |
| **HARMS2-AF, mean (SD)** | 7.79 (2.4) | 7.16 (2.5) | <0.0001 |

BMI, body mass index; SD, standard deviation.
*Sleep apnoea was not included in the HARMS2-AF score, limited to n=10,794 due to missing data
** To compare variables across case/control groups, chi-square tests were used for categorical variables, and t-test for continuous variables.

Table S5. Comparisons of risk prediction models for incident AF

| **Predictor** | **HR (95% CI)** | **p**-**value** |
| --- | --- | --- |
| **Roselli 2025 AF-PRS*** | 1.71 (1.59 - 1.84) | <0.0001 |
| **CHARGE-AF 2013** | 1.50 (1.38 – 1.63) | <0.0001 |
| **HARMS2-AF 2023**** | 1.32 (1.21 - 1.44) | <0.0001 |

AF-PRS, Atrial Fibrillation Polygenic Risk Score, CI, Confidence Interval; HR, Hazard Ratio.
* adjusted for age, sex, and top 10 genetic principal components
** Excludes sleep apnoea.

Table S6. Sex Stratification Cox models analysis for clinical risk scores.

| **CHARGE-AF** | | | |
| --- | --- | --- | --- |
| **Women (n=301 probable AF, n=6058 no AF)** | | **Men (n=327 probable AF, n=5785 no AF)** | |
| **HR (95% CI)** | **p**-**value** | **HR (95% CI)** | **p**-**value** |
| 1.43 (1.29 - 1.60) | <0.0001 | 1.58 (1.41 – 1.74) | <0.0001 |

AF, Atrial Fibrillation; CI, Confidence Interval; HR, Hazard Ratio.

Supplementary information for “Genomic risk prediction of incident atrial fibrillation in older individuals without prior cardiovascular disease”

**CHARGE-AF calculation**CHARGE-AF was calculated as the sum of each variable multiplied by its respective model coefficient:

Age x 0.5083 (per 5 years), white (yes) x 0.46491, height = 0.2478 (per 10 cm), weight = 0.1155 (per 15kg), systolic blood pressure x 0.1972 (per 20 mmHg), diastolic blood pressure x -0.1013 (per 10 mmHg), hypertension treatment (yes) x 0.34889, smoking (current) x 0.35931, diabetes (yes) x 0.23666, history of heart failure (yes) x 0.70127, and history of myocardial infarction (yes) x 0.49596.

**HARMS2-AF calculation**
The HARMS2-AF score is the sum of weighted variables as follows:

Hypertension: yes = 4, no = 0, Age: <60 = 0, 60-64 = 1, ≥65 = 2, BMI: <30 = 0, ≥30 = 1, Male Sex: Yes=2, no=0, Sleep Apnoea: yes=2, no =0, Smoking: current/previous =1, no=0, standard alcoholic drinks per week: <7 std. p/w=0, 7-14=1, ≥15=2.

**Baseline data:**Hypertension, diabetes, smoking and alcohol use, status was self-reported. Systolic and diastolic blood pressure was taken at randomisation and averaged over 3 measures. Aspirin (100 mg) and placebo use was randomised at baseline. Dyslipidaemia was defined as those taking cholesterol-lowering medications or serum cholesterol ≥212 mg/dL (≥5.5 mmol/L) or LDL>160 mg/dL (>4.1mmol/L). Thyroid stimulating hormone was measured from serum, using chemiluminescence microparticle immunoassay (Abbott Alinity ci, Abbott Diagnostics, Australia)
